# Common Interferences Removal from Dense Multichannel EEG Using Independent Component Decomposition

**DOI:** 10.1155/2018/1482874

**Published:** 2018-05-27

**Authors:** Weifeng Li, Yuxiaotong Shen, Jie Zhang, Xiaolin Huang, Ying Chen, Yun Ge

**Affiliations:** School of Electronic Science and Engineering, Nanjing University, Nanjing 210023, China

## Abstract

To improve the spatial resolution, dense multichannel electroencephalogram with more than 32 leads has gained more and more applications. However, strong common interference will not only conceal the weak components generated from the specific isolated neural source, but also lead to severe spurious correlation between different brain regions, which results in great distortion on brain connectivity or brain network analysis. Starting from the fast independent component analysis algorithm, we first derive the mixing matrix of independent source components based on the baseline signals prior to tasks. Then, we identify the common interferences as those components whose mixing vectors span the minimum angles with respect to the unitary vector. By assuming that both the common interferences and their corresponding mixing vectors stay consistent during the entire experiment, we apply the demixing and mixing matrix to the task signals and remove the inferred common interferences. Subsequently, we validate the method using simulation. Finally, the index of global coherence is calculated for validation. It turns out that the proposed method can successfully remove the common interferences so that the prominent coherence of mu rhythms in motor imagery tasks is unmasked. The proposed method can gain wide applications because it reveals the true correlation between the local sources in spite of the low signal-to-noise ratio.

## 1. Introduction

Electroencephalogram (EEG) collected from the scalp is the integration of the electrical activities of amounts of cortex neurons blurred by the skull [1]. Although it is widely accepted that EEG has the advantage of high temporal resolution, the spatial resolution remains as a problem [2]. To improve the spatial resolution, dense multichannel EEG (with more than 32 channels) and high-density EEG (with more than 128 channels) have gained more and more applications. However, the more the channels are used, the more the redundant information is involved. It directly results in the fact that the weak components generated from the specific isolated neural source are deeply concealed by the common components from the surrounding sources [3]. Moreover, these redundancies can lead to a spurious correlation/coordination between different brain regions while in fact little or none is present. It will greatly distort the result of the brain connectivity or brain network analysis, which becomes more and more popular [4–15]. Therefore, it is of great importance to unmask the isolated source-corresponding component from the originally collected signals with too much redundant information or common interferences.

Among multichannel EEG redundancy-removal methods, one representative is surface Laplacian reference scheme [16, 17]. After subtracting the average potential in the local neighborhood, the original signals referencing to one or two common locations are converted to referencing to the respective local one. Typically, the signal amplitude will greatly decrease, with the expected return of redundancy removal. The surface Laplacian reference scheme is theoretically simple and easy to implement. However, using the arithmetical mean within the neighborhood as the local reference may be a little bit rough, regardless of the conduction differences among the neighbor leads. In addition, great attention should be paid to the selection of the neighborhood.

Another representative is independent component analysis (ICA) [18, 19]. In fact, ICA has long been applied to EEG preprocessing [20–27] including electrooculography artifacts removal. Recently, Whitmore and Lin have succeeded in removing distal electrical reference as well as volume-conducted noises from local field potentials using ICA [25]. It greatly motivates us to step further, trying a more general common interference removal.

In the presented manuscript, we do not identify the source or the frequency of the common interference. Instead, we only assume that the common interference will affect the different channel most evenly and the mixing vectors keep constant during the whole experiment, regardless of the mental activities. In addition, by regarding both the common interferences and their transfer vectors as identical in the entire experimental circumstance, we adopt the component extracted from the baseline data. We validate the proposed method on BCI competition dataset 1 [28, 29]. It turns out that the method can successfully unmask the coherence in mu rhythm during a motor imaginary task. Since high-density EEG and brain connectivity or brain network are the trends in neuroscience, the proposed method can gain wide applications.

In the manuscript, we first describe the method in Section 2, and then in Section 3 the method is validated using simulation series as well as experimental data provided in BCI Competition IV, and finally results are discussed in Section 4.

## 2. Methods

The method includes three steps in order: independent components decomposition, the common interference identification, and removal and inverse transformation.

### 2.1. Independent Component Decomposition

Mathematically, given the independent *M* sources as **S** = (*s*_*i*,*j*_), *i* = 1,2,…, *M*, *j* = 1,2,…, *L*, in which *j* represents the sampling time index, the* N*-channel (*N* ≥ *M*) collected signal denoted as **X** = (*x*_*i*,*j*_), *i* = 1,2,…, *N*, *j* = 1,2,…, *L*, can be calculated as

(1) $$\begin{array}{c} X=AS, \end{array}$$

in which **A** is the* N*-by-*M* mixing matrix. Theoretically, each row of **A** represents a set of combination weights of the *M* different sources on the specific channel, and each column of **A**, denoted as ${\overset{⃑}{A}}_{j}$, reflects the relative impacts of the* j*th source on all the *N* different channels.

The independent component decomposition is to resolve (1) to obtain

(2) $$\begin{array}{c} S=A^{-1}X=WX, \end{array}$$

where **W** is called demixing matrix. Because neither **W** nor **S** is known a priori, the maximization of non-Gaussianity or minimization of mutual information principle is conventionally employed to approximate the **W** as well as **S** through iteration [12].

Herein, we adopt FastICA algorithm proposed by Hyvärinen [13] for independent component decomposition. The fixed-point iteration scheme as well as the maximum-negative entropy principle is employed to find the orthogonal rotation matrix **W** with the maximal non-Gaussian measure of the prewhitened data. And then the mixing matrix **A** can be calculated as

(3) $$\begin{array}{c} A=W^{-1}. \end{array}$$

### 2.2. Common Interference Identification and Removal

Subsequently, we try to identify and remove the common interference through analyzing the mixing matrix **A**.

The putative common interference component is assumed as a distal signal that has approximately same effect on all electrodes. In order to obtain local brain activities more accurately, these distal common interference components should be removed. To do this, the vector angles are calculated between each ${\overset{⃑}{A}}_{j}$ and a unit vector, and the smaller the angle is, the more uniform the impacts of the corresponding source (independent component) across channels are and the more likely the corresponding source is a common interference. We delete this source through setting the corresponding* k*th independent source *s*_*k*,*j*_, *j* = 1,2,…, *L* as 0, obtain the processed $\hat{S}$, and finally derive the deabundancy signals as

(4) $$\begin{array}{c} \hat{X}=A\hat{S} \end{array}$$

## 3. Experiments

### 3.1. Simulation

**Figure 1:**
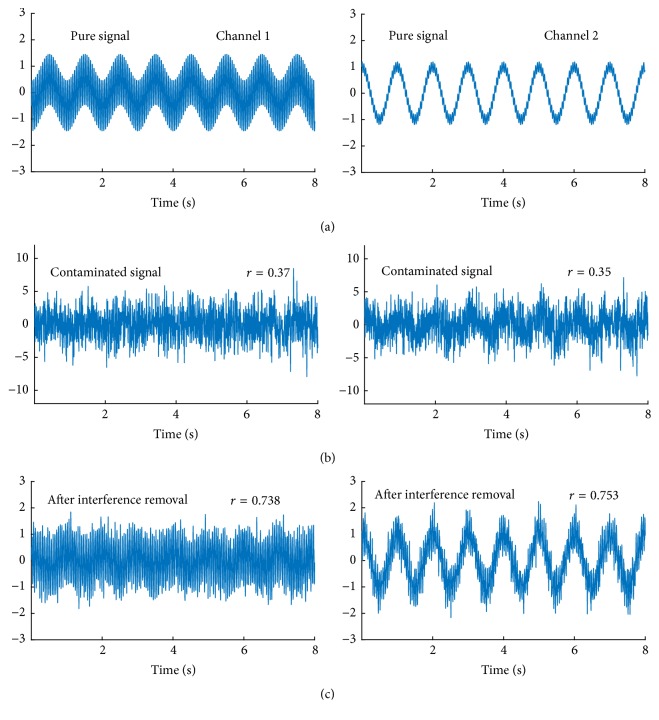
Simulation results ((a) is the pure signal, (b) is the contaminated signal, and (c) is the postprocessed signal. As we can see, although the noise is strong, the proposed method greatly improved the signal quality by doubling the correlation coefficient with the pure signal. Thus it validates the proposed method).

To validate the proposed method, we first applied it to simulation series. We define the three collected channel signals which are determined by three independent components, i.e., *s*_1_ = sin(2*π* × 10*t*), *s*_2_ = cos(2*πt*), and random Gaussian noises with *μ* = 0, *σ* = 10, and the mixing matrix $A=\left[\begin{array}{ccc} 1 & -0.5 & 0.19\\ 0.2 & 1 & 0.21\\ -0.4 & 0.4 & 0.2 \end{array}\right]$. As described in Section 2, the collected signals are derived by **X** = **AS**. The Gaussian component is deliberately set with great amplitude and is treated as the common interference. Theoretically, we can obtain the pure signal without common interference via setting the 3rd column elements as 0 s. We plot the pure signal of Channels 1 and 2 in Figure 1(a), and the collected contaminated signals in Figure 1(b). Then, we apply the proposed method to *X*. After common interference removal, signals of Channels 1 and 2 are plotted in Figure 1(c). To quantitatively evaluate the signal quality, we also calculate the linear correlation coefficient between the collected signals and the pure signals, both before and after common interference removal.

Figure 1 shows that the proposed method nearly doubles the correlation coefficient with the pure signals, and wave form also indicates the signal quality is greatly improved, even in such low signal-to-noise ratios.

### 3.2. Application to Scalp EEG

#### 3.2.1. Data Description

We apply the proposed method to the calibration data in dataset 1 of BCI Competition IV, provided by the Berlin BCI group [20, 21].

**Figure 2:**
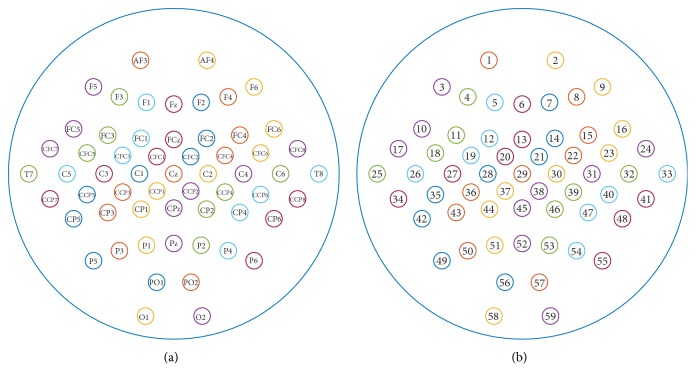
Lead locations for signals in dataset 1 ((a) presents the lead label, and (b) presents the lead number, in case we would refer to it in the manuscript).

This dataset includes three artificial data (#c, #d, and #e) as well as four data pieces recorded from 4 healthy subjects (#a, #b, #f, and #g) in motor imagery experiments. Each data includes 59-channel continuous EEG or artificial simulated EEG, with a sampling rate of 1000 Hz and high cut-off frequency of 200 Hz. To compress the data size, the provider downsampled the data to 100 Hz after low-pass filtering them with stopband edge frequency 49 Hz [21]. According to the data information, we plot the lead locations in Figure 2.

In each experiment, before the first cue was given, the very first duration of 16 s can be considered the baseline signal, and then 200 trials of cue-response with 8 s duration were followed. Each trial consists of 4 s cue and motor imagery task, 2 s blank screen, and 2 s fixation. Motor imagery can be movement of left hand, right hand, or feet, and for each subject two classes of motor imagery were chosen. The first 2.56 s sections beginning with the cue are used for the following analysis.

#### 3.2.2. Common Interference Removal

The baseline signals are firstly taken as original data to calculate the best orthogonal rotation matrix *W* and no more than 59 independent components *S* by FastICA [13]. The stopping criterion of FastICA is set as the minimum weight change of 10^−5^.

Although the brain activities related independent sources might be different between the baseline and task trials, both the common interference signal itself and its corresponding transfer vector are assumed to be identical in the entire experiment. Therefore, the putative common interference components calculated by the baseline signals can be extended to the following task state of the EEG treatment. That is, **W** is applied to task trial signals:

(5) $$\begin{array}{c} S^{task}=WX^{task} \end{array}$$

After deleting the common interference, we obtain the processed signal as

(6) $$\begin{array}{c} {\hat{X}}^{task}=A{\hat{S}}^{task} \end{array}$$

#### 3.2.3. EEG Results

**Figure 3:**
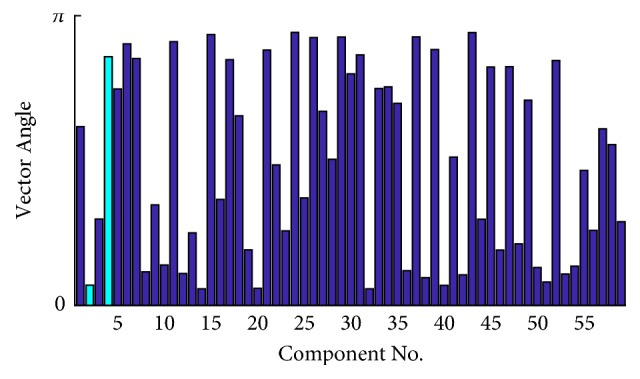
The vector angle of demixed independent component (the light blue marks the two components that are treated as common interference and removed).

We apply the proposed method to EEG. As a representative, we present the vector angle derived from #a in Figure 3, in which the light blue marks the two components treated as the common interference and then removed. As seen, these two components are not of the two smallest vector angles. However, we set an additional restriction that all elements in the mapping vector should be of the same sign. Therefore, in this case components 2 and 4 are determined as the common interference.

**Figure 4:**
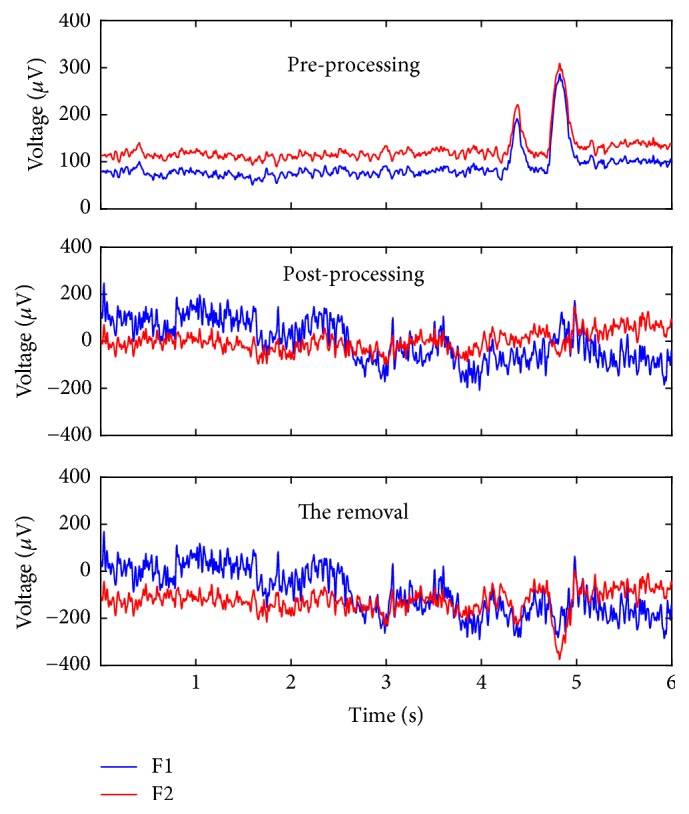
A representative EEG series result of subject #a. (Frontal EEG is usually contaminated by ocular and eye movement artifacts. This section includes two obvious ocular artifacts, occurring from 4.3 s to 5 s. After processing, the eye movement artifacts are successfully removed, and the correlation between F1 and F2 is alleviated)

We also examined the EEG series before and after the processing. As a representative, we plot two leads of subject #a in Figure 4. As shown in Figure 4, the original signals collected from leads 5 (F1) and 7 (F2) are highly correlated. And the eye movement artifacts are obvious, occurring from 4.3 s to 5 s. After processing, the correlation is alleviated, and the eye movement artifacts are removed.

**Figure 5:**
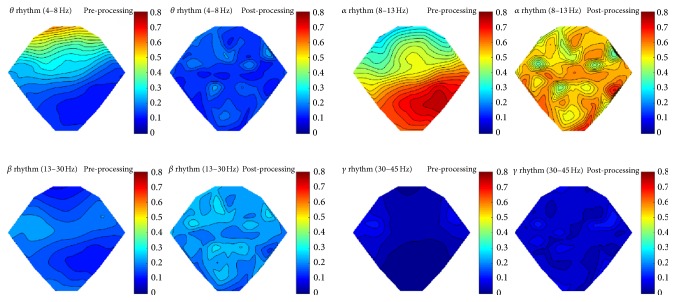
The relative power comparison between the original signal and the common interference removed signal. (Color represents the specific rhythm power relative to the power of the entire frequency band. As we see, after processing, the relative power reveals more distribution characteristics. It proves that we do uncover the intrinsic isolated neural activities, which were concealed by the strong common interference)

**Figure 6:**
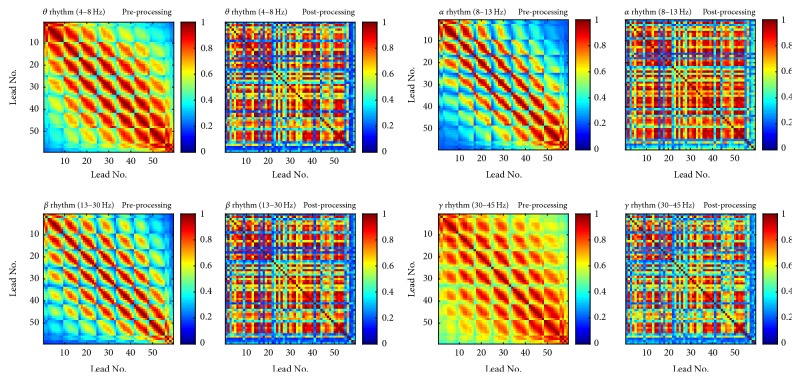
Coherence heatmap comparison between the original signal and the common interference removed signal. (As we can see, before processing, theta and gamma rhythm both present strong coherence for nearly all lead pairs. After processing, coherence differences among different pairs become obvious. In addition, the brighter lines parallel to the diagonal line diffuse to wider region after processing. It implies that after the common interference removal coherences between some far-away lead pairs become unconcealed and prominent)

It is difficult to provide an accurate signal quality evaluation, because we in fact do not know the “real” signal. However, we tried to calculate relative power as well as coherence and made comparison between the original signals (preprocessing) and processed signals with common interference removed (postprocessing). Taking the subject #a as an example, we present the relative power in Figure 5 and coherence heatmaps in Figure 6 for the commonly defined EEG rhythms.

As we can see from Figure 5, maps of the original signals have bigger connected regions, whereas after processing maps reveal more distribution characteristics. It proves that we do uncover the intrinsic isolated neural activities, which were concealed by strong common interference.

As we can see from Figure 6, for the original signal, the common interference imposes strong coherence on the leads in the same neighborhood. That leads to the brighter lines parallel to the diagonal line in the heatmap, which may conceal coherence between leads that are not close in location. However, after being processed by the proposed method, the bright neighborhood diffused, and coherences between some far-away lead pairs become unconcealed and prominent.

**Figure 7:**
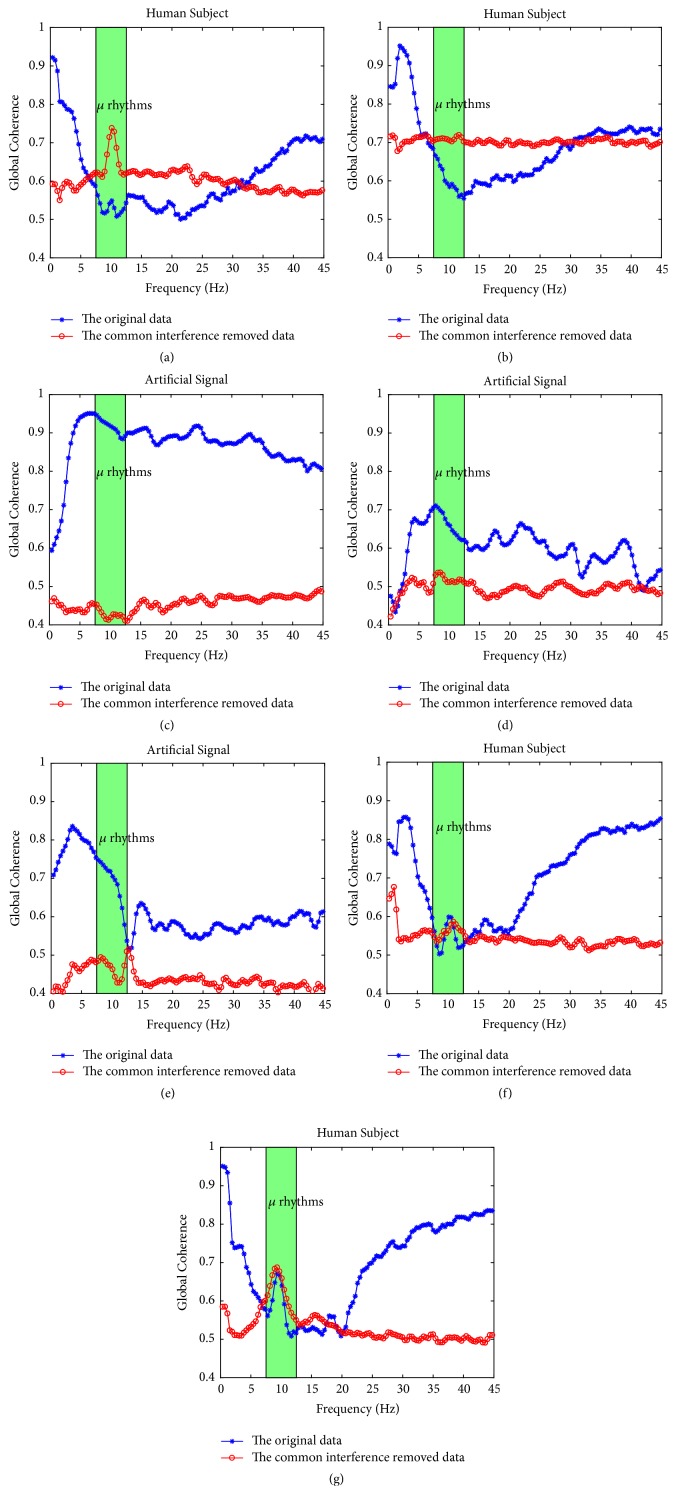
Comparison of the index of global coherence between the original and the processed signals. (The horizontal axis represents the frequency, and the vertical axis represents the index of global coherence. In the original data, all human subjects, i.e., (a), (b), (f), and (g), present high coherences in both low frequency band and high frequency band, which indicates a universal conductance induced consistency on scalp. However, as for the processed data, the high coherences in that two frequency bands are both suppressed while a coherence peak in mu rhythms (the green shade area) becomes prominent except for subject #b. It implies that although we did not mean to filter the specific frequency, the spurious high coherences caused by the common interferences are greatly alleviated. We cannot observe mu coherence in the artificial signals, i.e., (c), (d), and (e))

We further calculated an interesting index, i.e., the global coherence [3, 22], and made comparison. All global coherence results for the data sets 1 are presented in Figure 7, in which the horizontal axis represents the frequency, and the vertical axis represents the index of global coherence.

As shown in Figures 7(a), 7(b), 7(f), and 7(g), in the original data, all human subjects present high coherences in both low frequency band and high frequency band, which indicates a universal conductance induced consistency on scalp. However, as for the processed data, the high coherences in that two frequency bands are both suppressed while a coherence peak in mu rhythms (the green shade area in Figure 7) becomes prominent except for subject #b. It implies that although we did not mean to filter the specific frequency, the spurious high coherences caused by the common interferences are greatly alleviated. Meanwhile, the coherence in mu rhythms, which are intrinsically related to the motor imaginary, is unmasked. And as to the artificial signals in Figures 7(c), 7(d), and 7(e), we cannot observe mu coherence. Since these signals are artificial, we consider it reasonable. Therefore, the above results demonstrate that the proposed method is successful.

## 4. Discussions and Conclusion

Coherence is the equivalence of correlation in frequency domain. In active brains, correlation analysis in time domain is difficult because the EEG amplitude is very weak for desynchronization. In these cases, coherence is the appropriate substitute. However, whether in time domain or in frequency domain, the spurious correlation brought by the common interference imposes a big problem on unmasking the true cooperation between the weak neural sources. In the presented work, we propose an independent component decomposition based method; the two most crucial innovations include the following: (1) the angle between the mixing vector and the unitary vector rather than the frequency or morphology is used to identify the common interference; and (2) the independent component source and the mixing vectors derived from the baseline signal are applied to the following task signals. As to (2), since most EEG experiments are implemented in stimulus-locking paradigm, the proposed method can gain wide applications. In brief, the proposed method presents successful application in the motor imaginary EEG of BCI Competition IV and reveals the coherence peak in motor related mu rhythms.

## Appendix

The cross-spectral matrix **C** is calculated as

(A.1) $$\begin{array}{c} C_{ij}^{X}\left(\;f\;\right)=\frac{1}{K}\overset{K}{\underset{k=1}{\sum }}X_{i}^{k}\left(\;f\;\right)X_{j}^{k}{\left(\;f\;\right)}^{*}, \end{array}$$

in which *X*_*i*_^*k*^(*f*) and *X*_*j*_^*k*^(*f*) are the spectrums calculated from channels *i* and* j*, respectively, at frequency *f*. Then the cross-spectral matrix is singular value decomposed as **C** = **U****S****V**, where **S** is the diagonal matrix with each diagonal element, denoted as *λ*_*i*_, being an eigenvalue, and *λ*_1_ ≥ *λ*_2_ ≥ ⋯≥*λ*_*N*_. Finally, the global coherence is calculated as

(A.2) $$\begin{array}{c} {Coh}_{Global}=\frac{\lambda_{1}}{\sum_{i=1}^{N}\lambda_{i}}. \end{array}$$
